# Clinical retrospective study of dental implant removal: do patients who require implant removal desire implant prosthesis again?

**DOI:** 10.4317/medoral.23789

**Published:** 2020-10-09

**Authors:** Shintaro Sukegawa, Masato Saika, Ryo Tamamura, Keisuke Nakano, Kiyofumi Takabatake, Hotaka Kawai, Hitoshi Nagatsuka, Yoshihiko Furuki

**Affiliations:** 1 Department of Oral and Maxillofacial Surgery, Kagawa Prefectural Central Hospital, Japan; 2 Department of Oral Pathology and Medicine, Graduate School of Medicine, Dentistry and Pharmaceutical Sciences, Okayama University, Japan; 3 Department of Histology, Nihon University, School of Dentistry at Matsudo, Japan

## Abstract

**Background:**

This study investigated the causes of dental implant removal due to complications, and examined whether patients who had dental implant removal desired re-implant prosthesis treatments.

**Material and Methods:**

A retrospective case–control study was conducted on patients who had their dental implants removed. We investigated whether the removed dental implant was replaced with other implant prostheses. Age, sex, diabetes, smoking, implant site distribution, reason for implant removal, and blade and root-form implants were categorized as predictive variables. The outcome variable was desire for re-implantation or use of other prosthetic methods after implant removal. A logistic regression model was created to identify patient factors that could predict the re-implantation of dental prostheses after implant removal.

**Results:**

A total of 215 dental implants were removed from 143 patients. The most common reason for implant removal was peri-implantitis that was identified in 165 implants. After implant removal, re-implantation was performed in 98 implants (45.6%). Bivariate analyses showed that age, diabetes, implant type, and reason for implant removal were associated with the desire for re-implanted prostheses. The multiple regression model revealed that age, implant type, and reason for implant removal were associated with an increased desire for re-implant prostheses after implant removal.

**Conclusions:**

Re-implantation of prostheses after the removal of dental implants was desired by patients who were younger, had implants placed in the root form, and had implants removed due to prosthetic-related complications.

** Key words:**Dental implant removal, complications, implant prosthesis, retrospective study, re-implantation.

## Introduction

Titanium alloy implants have become an effective treatment modality for missing teeth (1). Although blade-type implants have been used in the past, this style of implant is rarely used at present due to the low survival rate that has been reported and associated with the formation of connective tissue around the implant (2,3). Unfortunately, patients with previously implanted blade-type implants continue to experience the need for implant removal due to complications (4). In contrast, the currently used osseointegrating root-form implants have been established as a promising alternative to prosthetic restoration with dental implants for lost teeth (5). The reliability and high success rate of dental implant treatments make the prognosis of implanted prostheses predicTable and facilitate safe intraoral maintenance (6), thereby increasing the number of patients being treated with dental implant prostheses.

The prognosis and reliability of implanted dental prosthetics are increasing and widespread. Dental implant treatments are often associated with complications such as peri-implant mucositis/peri-implantitis, which is an inflammatory disease, fracturing of the implant body, and complete loss of osseointegration, which can cause problems with the implant itself. A meta-analysis estimated the incidence of peri-implantitis at 12.8% (7). The causes of peri-implantitis are firstly bacterial (8) and secondly clinically induced (surgical, prosthetic and biomechanical) trigger factors (9). Surgical factors include implant malpositioning and inadequate bone augmentation, and prosthetic factors include excessive cement (10) and prosthetic design not adequate for proper hygiene and biomechanical factors include overload (11). Implant failures and complicated cases must be efficiently resolved (12). Unfortunately, the treatment of complications may entail removal of the dental implant. Current dental implants have a long-term prognosis, and complications may occur after implant placement. Long-term follow-up associated with complications often cannot occur at the same facility where the implant was placed. Therefore, the incidence and causes of dental implant removals associated with complications and the choice of replacement prostheses following implant removal remain unknown.

This retrospective case–control study investigated the causes of dental implant removal associated with implant complications and examined whether patients who experienced dental implant removal desired re-implant prosthetic treatments.

## Material and Methods

- Study design and sample

We designed and implemented a retrospective survey using the clinical, medical, surgical, and radiographic (panoramic radiography and/or computed tomography) records of patients who underwent dental implant removal surgery due to complications. The study population comprised patients treated by four maxillofacial surgeons in a general hospital practice setting. All implant removal surgeries were performed at a single general hospital (Kagawa Prefectural Central Hospital, Takamatsu, Japan) between April 2008 and March 2019.

Dental implant removal was indicated according to the complications that resulted in removal. The dental implants in this study were removed due to complications associated with previously surgically placed intraosseous implants. The clinical indications for removal included implants affecting the surrounding tissues due to inflammation that could not be improved or cases in which continuous prosthetic treatment was not possible. Inflammatory complications included peri-implantitis and maxillary sinusitis. Complications that required discontinuation of implanted prosthesis included nonremovable abutment screw fixture fractures, implant fixture fractures, and impossible prosthesis due to unspecified implant systems. Difficulties confirming the implanted prosthetic system included cases wherein the previous doctor’s office was closed, the doctor was unable to contact the clinic where the implant was placed, or the medical chart was discarded. The inclusion criteria for this study were as follows: (i) removal of dental implants, as defined above; (ii) availability of patients’ complete medical records for evaluation; and (iii) availability of predental implant removal radiographs or computed tomography images. The exclusion criteria were implant removal cases at the request of the patient without complications.

In some cases, patients requested re-implantation treatments for some removed implants but not for all removed implants. Therefore, this study examined implant restorations for all removed implant units. The design and methodology of this study were approved by the Ethics Committee of Kagawa Prefectural Central Hospital (approval no. 895).

- Predictive variables

The predictive variables for the study comprised factors that were considered to be convincingly related to dental implant removal and were classified as individual attributes, health status measures, and implant-related variables. Individual attributes included sex and age. Health status measures included the presence or absence of diabetes and the use of corticosteroids. Dental implant-related variables included implant site distribution, dental implant system, and reason for implant removal. The dental implant site distribution was classified as the maxilla and mandible, anterior, premolar and molar area, and left and right.

Dental implant systems were categorized into two types—blade and root-form. In this study, osseointegrated titanium implants were classified as root-form type implants.

The reasons for implant removal were categorized as inflammation-related complications and prosthetic complications. Inflammatory diseases included peri-implantitis and implant-related maxillary sinusitis, and prosthetic complications included broken implant fixtures, broken abutment screws, and impossible prosthesis due to unspecified implant systems.

- Outcome variables

We investigated whether the prosthesis used after the removal of a dental implant was an implant prosthesis or other prosthetic, such as dentures or bridges. The outcome variable was the desire for re-implantation or the use of other prosthetic methods after implant removal.

- Statistical analysis

Data were entered into a database using Microsoft Excel (Microsoft Inc., Redmond, WA, USA). Results were reviewed by an independent statistician. The database was transferred to JMP version 14.2 for Macintosh computers (SAS Institute Inc., Cary, NC, USA) for statistical analysis. For quantitative variables, normality was evaluated. If the quantitative variable was normal, parametric tests were performed, otherwise non-parametric tests were selected. For categorical variables, including sex, diabetes, use of corticosteroids, smoking, alcohol intake, maxilla/mandible, left/right side, implant system, and reason for implant removal, the Chi-squared test was used. If the expected frequency was <five, Fisher’s exact test was selected. All variables with *p* ≤ 0.05 were then entered into the logistic regression model. The logistic regression equation was used to calculate the adjusted odds ratios (AORs) of the included predictive variables with 95% confidence intervals (95% CIs). AORs and 95% CIs were calculated for each categorical variable. *P* values of <0.05 were considered statistically significant.

## Results

A total of 143 patients were included in this study, and 215 dental implants were removed from these patients. After the removal of dental implants, 98 individuals (45.6%) requested treatment with new dental implants.

- Reasons for dental implant removal

The removed dental implants comprised 174 root-form types and 41 blade types. The most common cause for dental implant removal was peri-implantitis, which was identified in 165 cases (127 root-form and 38 blade types), followed by broken implants in 21 cases (20 root-form and 1 blade type), broken abutment prosthetic screws in 11 cases (all root-form types), maxillary sinusitis in 12 cases (10 root-form type and 2 blade types), and impossible continuous prosthesis due to an unknown implant system in 6 cases (all root-form types) (Table 1).

- Prosthesis after implant removal

After implant removal, 98 implants (45.6%) were reinserted. Table 2 summarizes the bivariate relationships between the variables and the prosthesis after implant removal. The following variables were found to be significantly associated with complications (*p* < 0.05): age, diabetes, implant form, and reasons for implant removal.

We developed a multivariate logistic regression model that included the following candidate variables that were identified as being significant or nearly significant in the bivariate analyses (*p* < 0.05): age, diabetes, implant system, and reasons for implant removal. Age (AOR = 1.06, *p* < 0.0001), implant system (AOR = 5.87, *p* = 0.004), and reasons for implant removal (AOR = 3.74, *p* = 0.005) were significantly associated with an increasing desire for re-implantation of dental prosthesis after implant removal (Table 3).

**Table 1:**
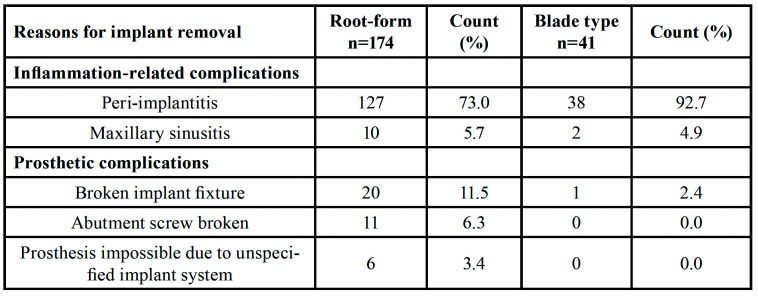
Reasons for dental implant removal.

**Table 2:**
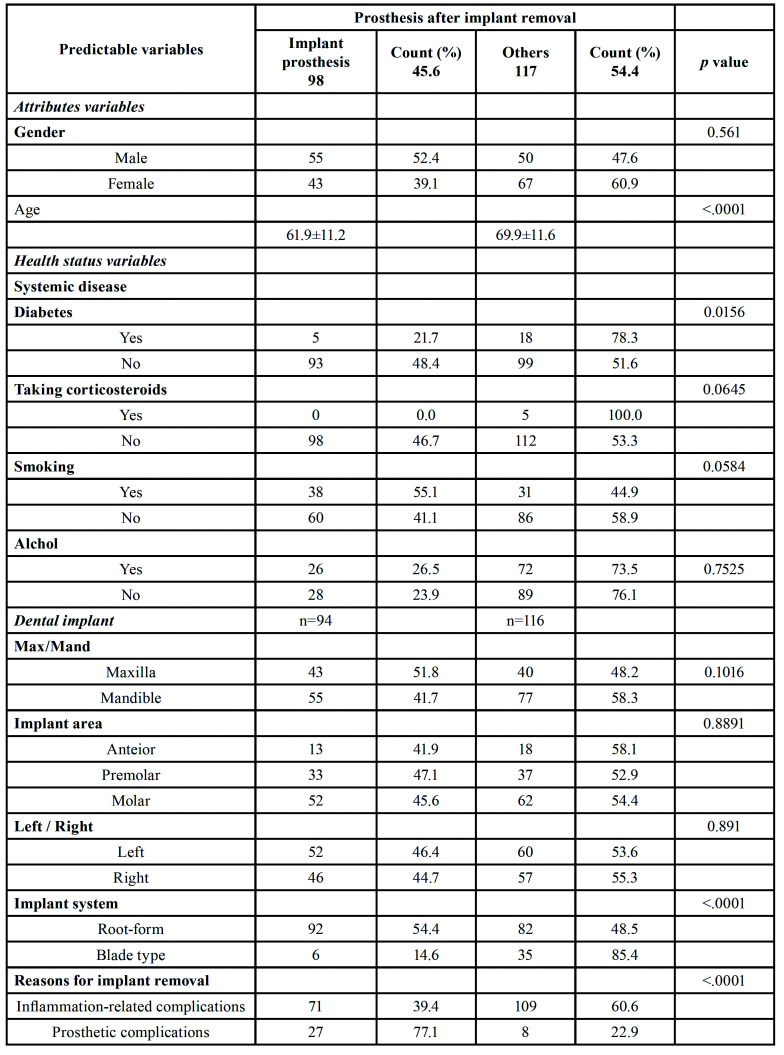
Factors associated with the desire for prostheses after implant removal in the bivariable analyses.

**Table 3:**
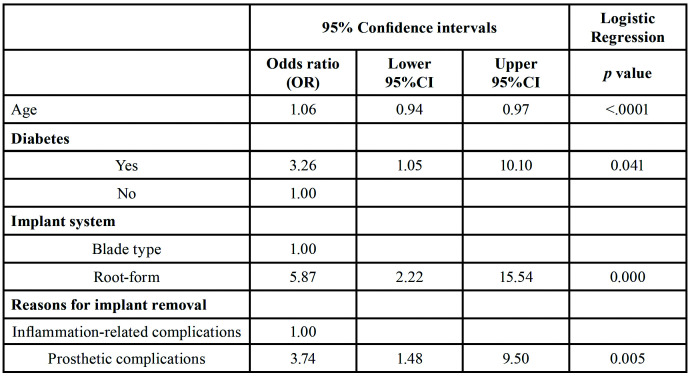
Logistic regression model.

Disscussion

Dental implants have several important advantages such as improved mastication efficiency and restoration of aesthetics and have become common prosthetic methods for dental treatment. Furthermore, several studies have reported high implant success and survival rates (13). Dental implant treatment is a highly reliable treatment; however, despite the low incidence of implant failure, failures occur occasionally, even with expert dentists. Therefore, it is important to understanding the causes associated with implant failure in clinical medicine. The reasons for dental implant failure typically include peri-implantitis, improper prosthetic construction and fit, and implant fracture due to overload arising from the load-bearing capacity of the surrounding bone (14). Implant failure causes can be categorized into inflammatory diseases and implant prosthesis-related problems. In our study, peri-implantitis was the most common reason for implant removal.

Peri-implantitis has been described as a pathological condition that occurs in functionally loaded tissues that surround dental implants and is characterized by mucosal inflammation and progressive marginal bone loss (15). The clinical features of peri-implantitis are enlarged periodontal pockets and bleeding from probing (16). It has been reported that blade-type implants are encapsulated with fibrous tissue, which has been proposed as a determinant of implantation failure due to the micromotion induced in the implant by the prosthesis (17). Consequently, this induces inflammation around the implant over the long term. Peri-implantitis in root-form implants represents a major biological complication and has been reported as the primary reason for late implant failure (18). Root-form implants do not become mobile unless osseointegration is almost completely lost. However, untreaTable peri-implantitis requires implant removal. Similar to the results of a previous study (19), untreaTable peri-implantitis was the most common reason for implant removal in the present study. Interestingly, patients who experienced insufficient occlusion due to implant inflammation and were associated with a long history of chronic inflammation tended not to desire re-implantation treatment.

In this study, the most common implant-related complications requiring implant removal were implant fracture and abutment screw fracture. A previous review reported that the prevalence of implant fixture fractures was <1% (0.08%–0.74%) (20), indicating that dental implant fixture fracture is a rare complication. However, dental implant fractures are unfortunately considered to be failures and require removal (21). The causes of this complication include severe bruxism, high occlusal force, mechanical trauma, implant material fatigue, and advanced bone loss, leading to reduced mechanical support surrounding the implant (22). The risk of dental implant fractures increases with the life of the implant (23). Dental implant fractures have been shown to increase the risk of continuing to bite for several years and have the potential for a certain number of complications. Abutment screw fractures are rare compared with dental implant fractures, with a prevalence of approximately 0.6% (24). The removal of an internally broken abutment screw is a time-consuming and challenging process due to poor visibility. Therefore, various methods have been reported for the removal of broken abutment screws, such as a low-speed bar converted into a flat-blade screwdriver (25), a fork-type rotating device combined with holes drilled in the center and the periphery of the damaged screw (26), and the use of an ultrasonic device (27). However, these methods can sometimes risk damaging the internal threads of the implant fixture. Consequently, the implant can become unusable. Furthermore, the recovery of the tightened abutment screw can be difficult depending on the fracture site (26), in which cases, the implants must be removed. These prosthetic-related complications were influenced by occlusion, and the development of these complications tended to indicate that the patients were adequately occluded. Interestingly, patients who required implant removal due to prosthetic-related complications were more likely to desire the replacement of implanted prosthetics.

Identification of the dental implant manufacturer and system is extremely important, particularly for maintaining a sTable prosthesis over a long period of time and for the removal of dental implants. When removal is necessary due to peri-implantitis or similar conditions and if the superstructure of the implanted prosthesis can be removed, a removal tool can be used (19). Removal tools are inserted into the fixture, allowing the fixture to be unscrewed and preserving the surrounding cortical bone. However, the removal of dental implants from unknown implant manufacturers was observed in approximately 4% of cases in this study. Patients may not be able to visit the same dentists for various reasons, including worsening general condition, transfer, migration to another country, and closure of dental clinics. Patients may also have difficulty in visiting the hospital for various reasons, such as deterioration of the patient’s general condition, transfer, migration to other country, and closure of dental clinics. Therefore, the identity of implant manufacturers should be made visible in XP images or a global implant sharing system should be developed to facilitate the removal of implanted structures in unknown systems (28).

The desire for re-implantation treatment after implant removal was an extremely interesting result in this study. Younger patients and those with root-form type implants and prosthetic complications often anticipated re-implantation after removal of the dental implant. In addition, non-diabetic patients were also more likely to desire re-implantation, although this factor did not show the strongest correlation in regression analysis. These results suggested that younger and healthier patients with satisfactory implant occlusion were more likely to desire a second implant treatment. Therefore, preservation of the alveolar bone should be prioritized in patients with factors demonstrating a strong correlation with the desire for re-implantation treatment. The desire for re-implantation treatment despite implant removal due to complications would provide such patients with satisfactory occlusal recovery. In contrast, re-implantation treatment may not be desirable if satisfactory occlusal recovery was not achieved or if the complications made the patient uncomforTable. Alveolar bone formation has been proposed to be a measure for improving the success rate of re-implantation surgery for late implant failure (29). Bone loss is one of the causes of failure, and surgical procedures that facilitate the maximum possible preservation of the alveolar bone is desirable when removing implants. Therefore, dentists and oral surgeons should be cautious about this aspect.

This study has two limitations. The first limitation was that only few cases of dental implant removal were observed, and a trend toward the re-treatment of dental implants has been previously reported (30). Therefore, the approaches to dental implant treatments may vary over time. In the present study, several superstructure implants were removed at other dental clinics, and the techniques applied by different dentists for implantation and prosthetic management were also diverse. However, the present study is useful because it is the first report to investigate the relationship between the complications of implant removal and the subsequent desire for re-implant prostheses. Large-scale surveys are anticipated in the future. The second limitation is the retrospective nature of this study, which increases the risk of bias. Some implants may have been removed by other dentists. Further investigations through long-term prospective studies are warranted in the future.

## Conclusions

Re-implantation of prostheses after the removal of dental implants was desired by patients who were younger, had implants placed in the root form, and required implant removal due to prosthetic-related complications. For patients who express a desire for re-implantation of prostheses after the removal of an existing implant, the implant must be removed using techniques that consider the subsequent implant placement.
